# Long-term stability and scale-up of noncovalently bound gold nanoparticle-siRNA suspensions

**DOI:** 10.3762/bjnano.10.248

**Published:** 2019-12-23

**Authors:** Anna V Epanchintseva, Julia E Poletaeva, Dmitrii V Pyshnyi, Elena I Ryabchikova, Inna A Pyshnaya

**Affiliations:** 1 Institute of Chemical Biology and Fundamental Medicine, Siberian Branch of Russian Academy of Science, Lavrent’ev av., 8, Novosibirsk, 630090, Russian Federation

**Keywords:** colloidal stability, gold nanoparticles, scale-up, siRNA delivery, siRNA duplex stability, therapeutic nucleic acids

## Abstract

Gold nanoparticles (AuNPs) are a platform for the creation of nanoconstructions that can have a variety of functions, including the delivery of therapeutic nucleic acids. We previously designed a AuNP/small interfering RNA (siRNA) nanoconstruction consisting of siRNA noncovalently bound on the AuNP surface and showed that this construction, when coated with a lipid shell, was an efficient vehicle for the delivery of siRNA into cells. The goal of the present work was to study the possibility of scaling up the synthesis of AuNP-siRNA and its long-term storage without loss of physicochemical characteristics and siRNA duplex integrity as well as siRNA surface density. Dynamic light scattering, transmission electron microscopy, UV–vis spectroscopy, and electrophoresis were used to study the effect of scaling up the AuNP-siRNA synthesis and long term storage of its suspension on physicochemical properties of the samples and integrity of the siRNA duplex. It was shown that a ten-fold increase in the volume of the reaction mixture decreased the surface density of siRNA by about 10%, which influenced the corresponding physicochemical characteristics of the AuNP-siRNA suspension. The storage of the AuNP-siRNA suspension at 4 °C for different times resulted in the formation of particle clusters of high colloidal stability as demonstrated by conventional methods. These clusters completely disintegrated when albumin was added, indicating that they are agglomerates (and not aggregates) of AuNP-siRNA. The AuNPs-siRNA nanoconstruction demonstrated integrity of the siRNA duplex and high stability of the siRNA surface density during storage for seven months at 4 °C. Thus, it can be concluded that it is possible to scale-up the synthesis of noncovalent AuNP-siRNA and to obtain a nanoconstruction possessing high stability in terms of physicochemical characteristics and siRNA surface density for a long period.

## Introduction

Drug delivery to cells is only one application of nanoparticles in biomedicine; however, it occupies an important place in scientific research. The delivery of therapeutic nucleic acids (TNAs) is of particular importance due to their operation at the genomic level, and various nanoparticles (NPs) have been studied as carriers of TNAs (i.e., metallic, lipid, polymer, and peptide NPs, and their diverse compositions). Significant attention has been paid to gold NPs (AuNPs) due to their unique physical and chemical properties, where resent results are summarized and analyzed in recent comprehensive reviews [1–3]. The use of AuNPs as a vehicle for TNAs has many advantages including the opportunity to directly observe the site of drug location and monitor its movement in a cell, thereby making AuNPs particularly attractive for TNA studies, since different TNAs operate in cell different areas [1,4–5].

Small interfering RNA (siRNA) are considered to be a powerful tool for silencing the protein synthesis to improve abnormal protein production caused by genomic disorders or diseases [6–8]. Different variants of AuNP-based nanoconstructions bearing siRNA have been published, and most of them were created by applying the layer-by-layer principle [9]. The core of these structures usually consists of a AuNP, where siRNA is covalently bound to the NP surface through the thiol group, and the core is covered with a lipid or polymer shell [10–13]. Covalent bonding of siRNA to the surface of AuNPs is carried out in two stages: (1) covalent attachment of one chain of a duplex; (2) hybridization of the second chain with the first one. It should be noted that the efficiency of hybridization of the second chain with the first chain already covalently attached to the AuNP is not complete, it is about 50% [13]. The density of siRNA covalently attached to the AuNP surface varies from 30 to 100 molecules per 1 NP [12,14]. The covalent binding of siRNA to AuNPs raises another important question concerning desorption of siRNA chains from the NP surface since the implementation of siRNA function in cells strictly requires the presence of both strands [15]. None of the works published to date give information about the cleavage of the covalent bond between siRNA and AuNPs and preservation of the siRNA duplex inside the cell.

Previously we designed the construction (AuNP-siRNA) based on 12–14 nm AuNPs that contained noncovalently bound siRNA, 21 bp (base pairs), and determined the synthesis and storage conditions that provide stability of the construction. Using fluorescent labeling, we defined a range of siRNA surface density (40–160 molecules/1 NP) and identified the value (about 100 molecules per 1 NP) corresponding to the most stable coupling of siRNA to AuNPs. Then, we showed desorption of both strands of the siRNA duplex in the artificial cytosol and serum solutions [16–17]. In that way, we firstly obtained a new kind of AuNP-based construct containing noncovalently attached siRNA. Currently, noncovalent binding to AuNPs has been reported only for single-chain nucleic acids [18–21].

Then, we used AuNP-siRNA as a core for nanoconstruction by covering this with a lipid envelope and doping with an amphiphilic peptide. The construction was shown to penetrate cells that consistently expressed the green fluorescent protein (GFP) and effectively suppress the synthesis of GFP mRNA, evidencing the siRNA noncovalent attachment to AuNPs [17]. Our studies demonstrated that noncovalent binding of siRNA to the AuNP surface is a promising approach to the creation of theranostic nanoconstructions because this bond maintains siRNA in its physiological (functional) form and does not alter the properties and advantages of gold nanoparticles.

The stability and storage duration of the nanoconstructions is important for their use in research and technology. The important properties of the nanoconstructions may be compromised during storage because of desorption and degradation of molecules, attachment of extra molecules from the incubation medium, and irreversible aggregation of the particles. Previous studies concerning vehicles for TNAs describe their therapeutic effect but not their stability and storage conditions [12–14,20–21]. The storage problem of the final nanostructures with a biomolecular shell seems quite complex because of the high lability of many proteins, polymers, and lipids. The synthesis and storage of significant quantities of core materials for multi-level nanoconstructions could be an approach to solving this problem. Therefore, we decided to determine whether it is possible to scale-up the synthesis and to store a core of nanoconstructions with an example of noncovalent AuNP-siRNA without loss of their physicochemical properties and the integrity of siRNA duplexes for an extended period. To do this, we analyzed the colloidal stability of the AuNP-siRNA suspensions during storage by gel electrophoresis and dynamic light scattering, studied the samples by transmission electron microscopy (TEM), determined spectral characteristics of the AuNP-siRNA, and monitored the preservation of the siRNA on the AuNP surface by electrophoresis and measured the fluorescence intensity of the labeled sense siRNA strand.

The study demonstrated the long-term storage capability of the noncovalent AuNP-siRNA nanoconstruction without loss of their physicochemical properties and the siRNA duplex integrity over the period of 7 months.

## Results and Discussion

### Scaling up of the AuNP-siRNA synthesis

The development of new drugs involves the study of their specific activity in cell cultures and animals, which requires the preparation, storage, and use of significant quantities of tested substances [22]. Significant quantities of the preparation can be obtained by (1) increasing the concentration of the initial components or (2) increasing the volume of the reaction mixture while maintaining the concentration of the components. In this work, we studied the second approach since we have previously found that the surface density (number of siRNA molecules per one AuNP), which is an important indicator of the quality of the AuNP-siRNA preparations, decreases during concentration [17].

The object of our study was spherical AuNPs bearing noncovalently bound siRNA. Previously [17], we showed that the density of siRNA on the surface of AuNPs depends on the salt concentration in the aqueous solution and can reach 150 molecules per one particle. In this study we used a surface density of about 100 molecules of siRNA per 1 AuNP (102.5 ± 3.5) which provided better stability of the AuNP-siRNA construction.

We studied the possible effect of a ten-fold increase in the volume of the reaction mixture on the physicochemical characteristics of the AuNP-siRNA suspension (×1 and ten-fold volume ×10 samples).

**Figure 1:**
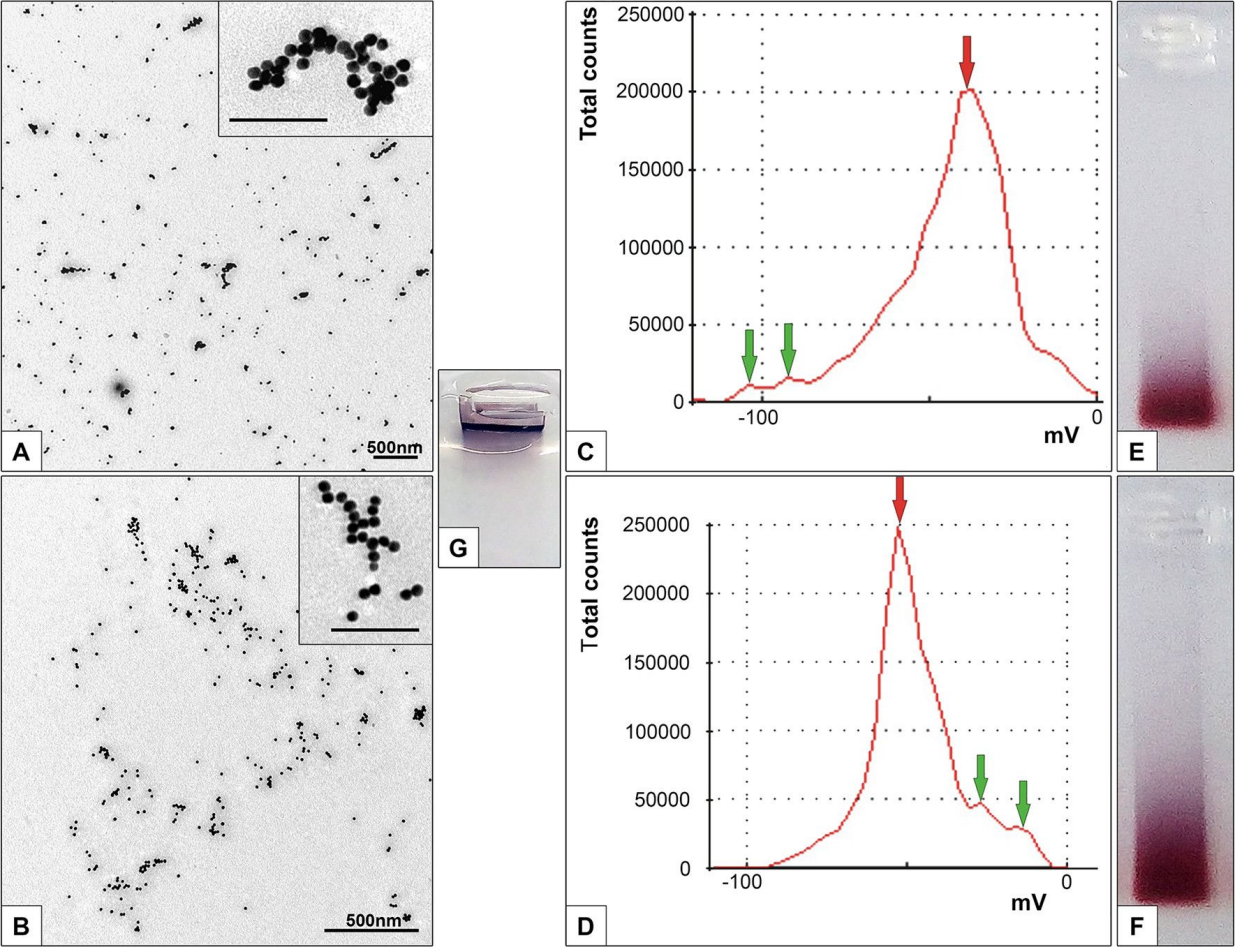
Physicochemical characteristics of freshly prepared AuNP-siRNA suspensions. TEM images of samples ×1 (A) and ×10 (B) adsorbed onto the grid. The inserts show enlarged examples of unusual “figures”. The scale bars in the inserts correspond to 100 nm. Distribution of the zeta potential values in samples ×1 (C) and ×10 (D) are also given. Red and green arrows show the major and minor peaks, respectively. The images of gels after electrophoretic analysis of samples ×1 (E), ×10 (F), and initial unmoved AuNP (G) (the samples were deposited on the gel in the same amount) are given as well.

In the TEM images, the freshly prepared AuNP-siRNA particles had a spherical shape and Au core diameter of 12.85 ± 0.35 nm, (Figure 1A,B), which corresponded to the size of the initial citrate AuNPs. Sample ×1 (Figure 1A) was highly dispersed, and the particles were arranged on the supporting film as individual particles or in short chains. The particles were separated by a clearly visible space of 2.09 ± 0.2 nm. Some chains formed unusual “figures”, but the space between the particles was always preserved, which obviously indicated the presence of the siRNA layer. In sample ×10 (Figure 1B) the chains and “figures” were observed more often. It should be noted that no aggregates/agglomerates of AuNP-siRNA (as shown on Supporting Information File 1, Figure S1) were observed in both samples.

The increase in the volume of the reaction mixture did not significantly affect the hydrodynamic AuNP-siRNA diameter, which was 33.39 ± 1.1 nm for sample ×1 and 37.51 ± 1.65 nm for sample ×10. The polydispersity index values (PdI) for sample ×1 and ×10 was 0.266 ±0.025 and 0.304 ± 0.010, respectively, thus indicating the homogeneity of the suspensions [23].

The main part (≈90% of the average value of the zeta potential) of the AuNP-siRNA particles in samples ×1 and ×10 had a charge of −38.85 ± 2.66 mV and −49.42 ± 2.07 mV, respectively (the main peaks in Figure 1C,D). The seed AuNP-siRNA (citrate) AuNPs had a zeta potential value of −33.6 ± 2.0 mV, indicating that attachment of siRNA influenced this value. The analysis of the zeta potential values of samples ×1 and ×10 revealed changes in the ratio between particles with different surface charge; this is clearly illustrated by the shape of the curves (Figure 1C,D; Supporting Information File 1, Table S1). At the same time, there were the particles with a charge value of −78.84 ± 10.85 mV (sample ×1) and −20.59 ± 8.04 mV (sample ×10), which was 9–11% of the average value of zeta potential. The average zeta potential value depends on the total charge of siRNA molecules associated with AuNPs, and the profile of the zeta potential curve (Figure 1C,D) reflects the presence in the sample of differently charged particles that carry the different number of the siRNA molecules. The results show that sample ×10 contains a share of AuNP-siRNA particles with a charge significantly below the average.

The analysis of the same amount of AuNP-siRNA sample by gel electrophoresis confirmed the presence of particles with a charge significantly below the average in sample ×10 (blue color track in Figure 1E,F). For comparison, we presented the electropherogram of the initial bare gold nanoparticles (Figure 1G).

The density of the siRNA molecules on the AuNP surface is an indicator of the efficiency of the nucleic acid transporter: the higher the density, the less amount of the preparation can be administered, thus reducing the toxic effect of siRNA [24].

In this work, we prepared AuNP-siRNA according to previously established regulations, and noncovalent sorption of the siRNA molecules on AuNPs was shown to be reversible [16,25]. The surface density of siRNA was calculated from the measurements of the fluorescence intensity of the Cy5.5 residue attached to the sense strand of siRNA. The values of the surface density of siRNA in freshly prepared samples ×1 and ×10 differed, i.e., they were 119.5 ± 2.9 and 102.5 ± 3.5 of the siRNA molecules per one AuNP, respectively. It can be assumed that this difference corresponds to the presence of about 10% of particles with a zeta potential of −21 ± 8 mV in sample ×10 (the average zeta potential value was −49.42 ± 2.07 mV). The results indicate that the observed changes in the physicochemical characteristics of the AuNP-siRNA suspension when scaling its synthesis are due to differences in the binding of siRNA to AuNPs. About 10% of the completed particles in sample ×10 have a lower surface density, which in turn determines the difference in the electrophoretic mobility of samples ×1 and ×10 as well as values of the zeta potential and the shape of their curves.

The only possible reason for the change in the formation of AuNP-siRNA with an increase in the volume of the reaction mixture is a geometric factor. Indeed, the formation of AuNP-siRNA is controlled by the surface area of the reactor (we used 1.5 mL, Eppendorf safe lock tubes, #0030120086). In our experiments, a ten-fold increase in the volume of the reaction mixture led to an increased surface area by a factor of 4.3.

**Figure 2:**
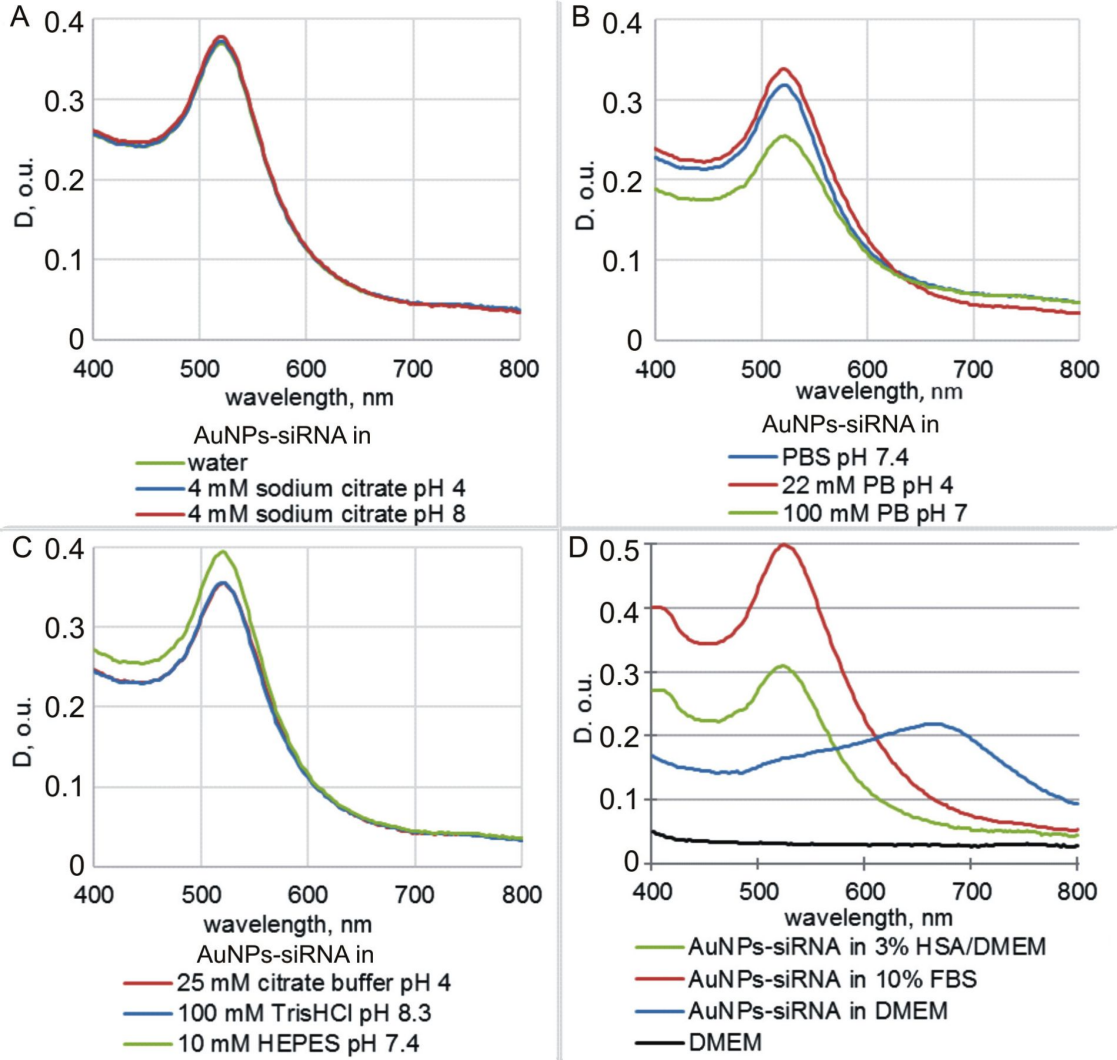
Optical extinction spectra of AuNP-siRNA in various solutions. The solvents are given in figures. We used samples with different concentrations to separate the curve maximums for clear visualization.

The incubation of the components in different media is often required when creating complicated nanoconstructions. We tested the stability of the noncovalent AuNP-siRNA in various solutions, including buffers with different pH values by analyzing the optical absorption spectra in the range of 400–800 nm (Figure 2). We did not observe a wide peak in the range of 650–700 nm, which would indicate nanoparticle aggregation. This peak appeared only in the case of mixing of the AuNP-siRNA suspension with the DMEM culture medium (Figure 2D) as was shown in [16]. The data indicate the stability of the noncovalent AuNP-siRNA in a wide range of pH (4–8.5) and in media of different chemical composition.

The analysis of the AuNP-siRNA suspensions by the standard methods for studying nanoparticles [26–27] showed that the freshly prepared suspension is homogeneous and has high colloidal stability in various solutions, except for the DMEM medium. A ten-fold increase in the reaction volume led to a decrease of about 10% in the surface density of AuNP-siRNA nanoconstructions, which caused changes in the corresponding physicochemical characteristics of the suspension. The increase in the volume of the reaction mixture may seem insignificant; however, this technique allowed us to use only one sample for transfection of 1,600,000 cells. Obviously, the use of the same sample of the preparation reduces the experimental error [17].

### Effect of storage duration on physicochemical properties of AuNP-siRNA suspension

The ability to store semi-finished products when creating TNA-based preparations is undoubtedly useful for obtaining large amounts for research and testing. However, this problem concerning nanoconstructions of TNA and metal nanoparticles is not covered in the literature, and began the study of this issue starting with a short period of 24 h. We focused on studying the main signs of preparation spoiling, aggregation of AuNP-siRNA and disruption of siRNA molecules (loss of duplex integrity).

The clumping of particles in the suspension can be reversible or irreversible, which leads to the formation of agglomerates or aggregates, respectively, according to the definition of the IUPAC International Committee [28]. All existing methods for the registration of aggregation/agglomeration of particles in colloidal suspensions are indirect and give approximate results. For the most accurate assessment of the process, it is recommended to use a set of methods although it is necessary to consider their limitations [26–27]. We evaluated the clumping of AuNP-siRNA in samples ×1 and ×10 during storage for 24 h and eight days at 4 °C by the methods of TEM, DLS, and electrophoresis.

**Figure 3:**
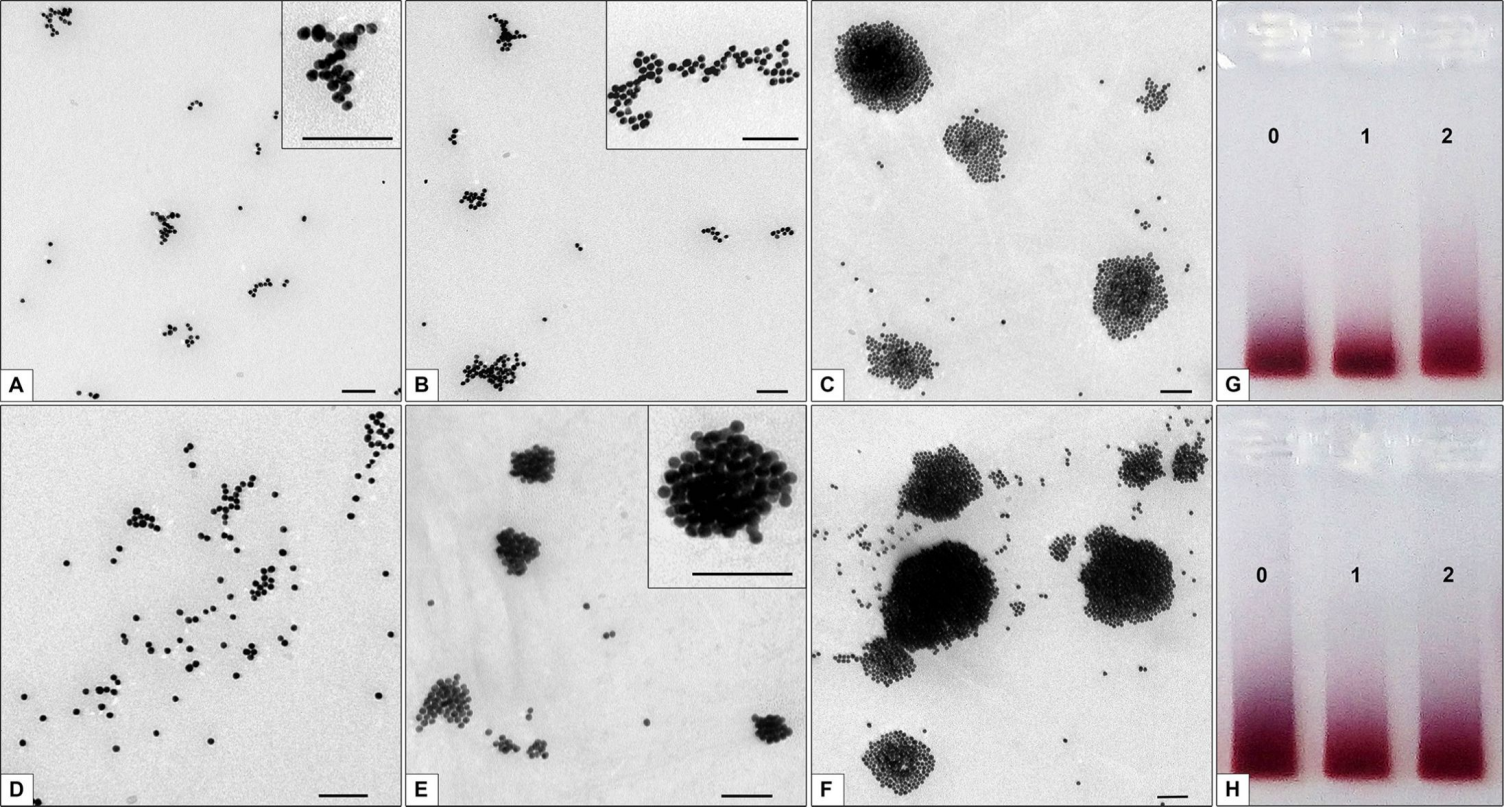
Clumping of AuNP-siRNA during storage for eight days at 4 °C. Upper row, sample ×1; lower row, ×10. (A) and (D), freshly prepared samples; (B) and (E), samples after one day of storage; (C) and (F), samples after eight days of storage. The inserts show figures and clusters at higher magnification. Scale bars correspond to 100 nm. Images of gels after electrophoretic analysis: (G) and (H), samples ×1 and ×10, respectively; lanes: 0, freshly prepared samples; 1, storage for one day; 2, storage for eight days.

The storage of sample ×1 for 24 h led to an increase in the number of the AuNP-siRNA chains and the appearance of loose branched “figures” of different shapes (Figure 3B) compared with the freshly prepared suspension (Figure 1A and Figure 3A). The appearance of rounded clusters of AuNP-siRNA with a diameter of not more than 200 nm was a consequence of aggregation/agglomeration of particles over the course of one day (Figure 3E). The particles in the clusters were arranged in several layers and were distinguishable. The scanning probe image processor (SPIP) analysis of the TEM image showed a predominance of clusters with a size of up to 100 nm in samples ×1 and ×10 (85–90% of the total number of clusters); about 10% of clusters had a size of 100–200 nm. Thus, the TEM study showed a tendency towards clumping of the AuNP-siRNA during storage for 24 h, and this process was more pronounced in samples ×10. The DLS study did not reveal significant changes in the characteristics of AuNP-siRNA samples ×1 and ×10 after a day of storage. The values of the hydrodynamic size of the particles were 40.14 ± 3.17 nm and 36.8 ± 4.17 nm, respectively. The PdI values were 0.266 ± 0.025 and 0.304 ± 0.010 (Supporting Information File 1, Table S2).

After eight days of storage, the TEM images of the AuNP-siRNA samples ×1 and ×10 showed numerous single particles and “figures” or chains uniformly distributed on the grid. In sample ×1, we observed rounded clusters of AuNP-siRNA (Figure 3C), which were single-layered or multi-layered in the center. In sample ×10, clusters were more frequent and reached a larger size (Figure 3F). The structure of the AuNP-siRNA different clusters are presented in Supporting Information File 1, Figure S1. According to the SPIP analysis of samples ×1 and ×10, the number of clusters with a diameter of more than 100 nm increased in both samples; clusters with a diameter of 100–200 nm accounted for 19% and 21% of the total, respectively. Sample ×10 was distinguished by the presence of 15% of clusters with a diameter of 200–300 nm (5% in samples ×1) and 10% of clusters with a diameter of 300–500 nm, which were absent in sample ×1. Thus, the storage of the AuNP-siRNA suspensions of for eight days led to clumping of particles, more pronounced in sample ×10.

The DLS study showed no significant changes in the values of hydrodynamic size and PdI for AuNP-siRNA after eight days of storage (Supporting Information File 1, Table S2). The electrophoretic analysis also revealed no significant changes in the state of samples ×1 and ×10 during this storage (Figure 3G,H).

The results showed the possibility of storing the AuNP-siRNA samples ×1 and ×10 for eight days at 4 °C without loss of their physicochemical properties. The next stage of the work was to examine the possibility of longer storage, up to four weeks, which was studied using only sample ×10 because sample ×1 evaporated after ten days of storage.

**Figure 4:**
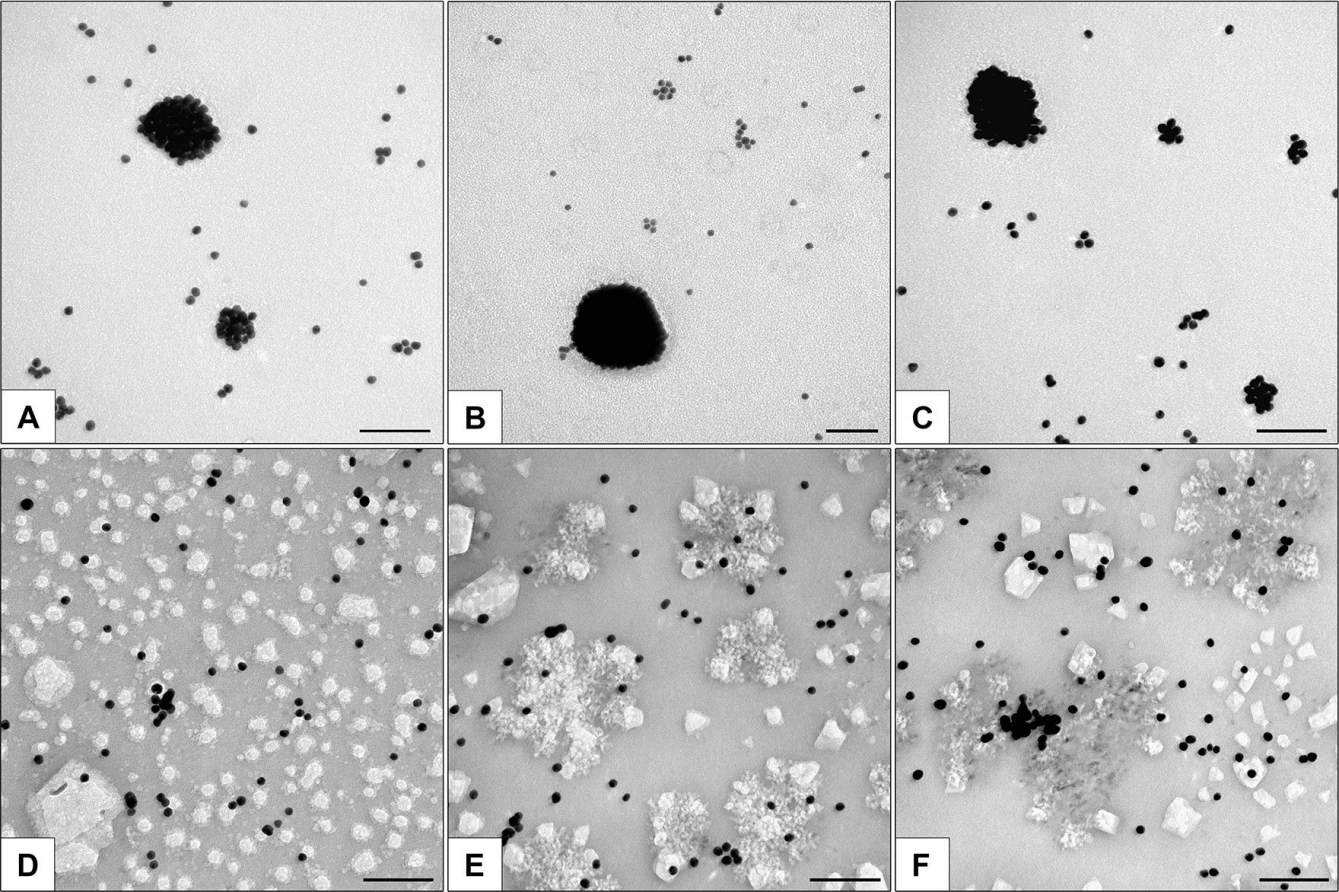
Clusters of AuNP-siRNA in samples ×10 after two (A), three (В), and four (С) weeks. The same samples after the addition of 3% human serum albumin (HSA) in the DMEM medium (D, E, F). D–F – contrasting with uranyl acetate. Scale bars correspond to 100 nm.

**Figure 5:**
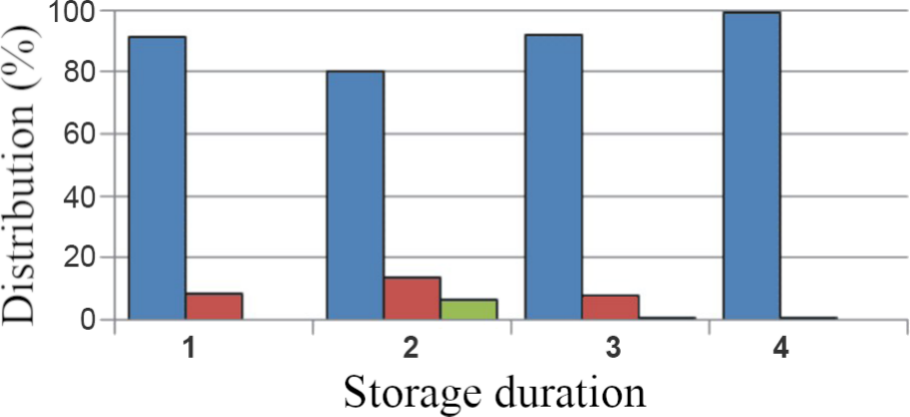
Data from the SPIP analysis. Distribution (%) of AuNP-siRNA clusters by size illustrating the formation of the AuNP-siRNA clusters during storage for two weeks (1), three weeks (2), four weeks (3) and 7 months (4) according to the SPIP analysis. The blue color corresponds to sizes of 20–100 nm; red color – to sizes of 100–200 nm and green – 200–400 nm.

According to the TEM data, the storage of sample ×10 for two, three, and four weeks led to the appearance of a numerous rounded dense clusters of different sizes in the AuNP-siRNA suspension (Figure 4A–C). We also observed numerous remaining single particles, as well as chains and “figures”. Interestingly, many clusters of the B and С types appeared in the sample stored for four weeks (Supporting Information File 1, Figure S1), which were absent earlier. The SPIP analysis showed that the main portion of the clusters was represented by small ones (<100 nm); the majority of the large clusters was observed after three weeks of storage (Figure 5). The images of the suspensions after two and four weeks of storage did not differ significantly.

The results show that the AuNP-siRNA suspension demonstrates a dynamic nature during four weeks of storage in that the size and structure of the clusters varied. Interestingly, the largest variation in the size of the clusters was observed after three, not four, weeks of storage. Similar results were obtained using the DLS method, i.e., the hydrodynamic diameter values of the AuNP-siRNA particles after two, three, and four weeks of storage were to 38.24 ± 1.75 nm, 43.03 ± 2.20 nm, and of 38.45 ± 1.75 nm, and the PdI values were 0.286 ± 0.005, 0.326 ± 0.006, and 0.407 ± 0.041, respectively. The formation of the AuNP-siRNA clusters during storage may be an indicator of a decrease in the quality of the preparation if the clusters are aggregates. Aggregation of nanoparticles in biological media is one of the serious causes of colloidal stability of the suspension. Therefore, the formation of aggregates should be carefully monitored [26–27].

The methods used in this study do not make it possible to differentiate aggregates from agglomerates. To prove the reversibility of the clumping of AuNP-siRNA, we used the known property of albumin to increase the dispersion of AuNP suspensions [29]. We added 3% human serum albumin (HSA) (corresponding to 10% serum commonly used in the cell cultivation) to samples ×1 and ×10 (freshly prepared and stored for one or eight days) before their examination by TEM and to sample ×10 stored for two, three, and four weeks. The similarity of the results for all samples were very impressive, i.e., all clusters disappeared, the particles were separately located on grids, and occasionally there were short chains. We observed the disappearance of the AuNP-siRNA clusters (Figure 4D,E,F) even after the addition of 3% albumin to the samples in the DMEM medium, which promoted aggregation of AuNP-siRNA as shown in Figure 2. The hydrodynamic parameters of the AuNP-siRNA particles after the addition of albumin were impossible to evaluate because of the influence of albumin. Thus, the storage of the AuNP-siRNA samples for four weeks leads to the reversible formation of agglomerates, which indicates no deterioration in the quality of the preparation.

**Figure 6:**
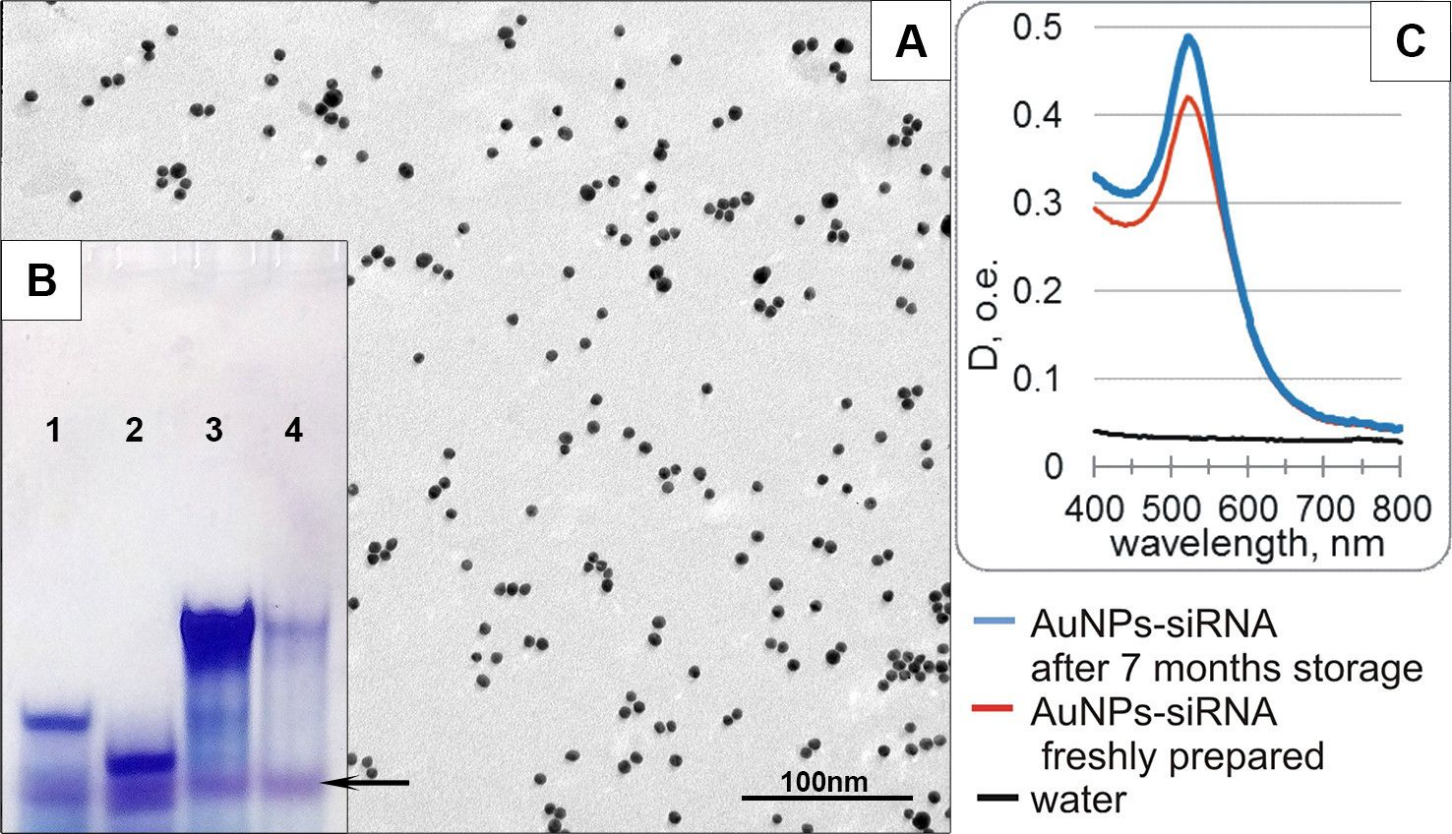
(А) AuNP-siRNA suspension (sample ×10) after storage at 4 °С for seven months. (В) Electrophoretic analysis of siRNA desorbed from the surface of AuNPs after storage for seven months (lane 4). Control lanes: 1, antisense strand; 2, sense strand; 3, siRNA duplex. Difference in intensity of bands (lanes 4 and 3) is related to different concentration of siRNA in samples (concentration of desorbed from AuNPs was four times less than control). Arrow shows BrPh dye. The electrophoretic analysis was carried out in 15% native polyacrylamide gel. (C) Optical extinction spectra of fresh AuNP-siRNA (red line) and after 7 months of storage (blue line).

We studied the characteristics of the ×10 AuNP-siRNA samples, which were stored at 4 °C for seven months. To our surprise, the suspension showed high dispersion in TEM and contained individual particles, short chains, and figures (Figure 6A). The SPIP analysis showed no clusters larger than 100 nm (Figure 5). The hydrodynamic size of the particles was 40.3 ± 3.7 nm and the PdI value was 0.449 ± 0.006, obviously due to the large number of small clusters. Optical spectra of the samples stored for seven months did not differ from the spectrum of freshly prepared suspensions (Figure 2, Figure 6C, Supporting Information File 1, Figure S2). The suspension showed stability in different media.

In general, the results indicate high colloidal stability of the AuNP-siRNA suspensions, i.e., there was no aggregation, and all the emerging AuNP-siRNA clusters were agglomerates, which easily disintegrated after the addition of albumin. This demonstrated the possibility of long-term storage (up to seven months) of the AuNP-siRNA suspensions without loss of their physicochemical characteristics and clearly indicates the stability of nanoconstructions obtained by the noncovalent binding of siRNA to AuNPs.

### Effect of storage duration on siRNA integrity in AuNP-siRNA suspensions

The storage of samples ×10 for four weeks (Supporting Information File 1, Table S3) and even seven months did not affect the value of the surface density of siRNA, which ranged from 102.5 ± 3.5 to 101.8 ± 0.5 siRNA molecules per one AuNP. This fact strongly indicates the high affinity of the noncovalent binding of siRNA to the surface of gold nanoparticles.

The integrity of siRNA in the AuNP-siRNA was examined after storage of the suspension for three weeks and seven months by the treatment with dithiothreitol (DTT) for the desorption of siRNA from the nanoparticle surface, followed by the analysis by gel electrophoresis (Figure 6B). We did not observe the separate strands of siRNA in the lane corresponding to siRNA desorbed from the surface (Lane 4), which indicated that siRNA in the nanoconstructions does not degrade during storage, and both strands of siRNA are desorbed from the surface in the equimolar ratio. Thus, the noncovalent attachment of siRNA to the AuNP surface provided the binding stability and the integrity of the siRNA duplex for seven months.

Our studies of the noncovalent nanoconstruction AuNP-siRNA, which we have been conducting for several years, showed that they could be used as a core for lipid-enveloped nanoconstructions to effectively deliver siRNA into the cells. The AuNPs are able to carry up to 150 siRNA molecules per one particle [17]. Both strands of siRNA are capable of desorbing from the surface in biological media thus retaining their native properties and the ability to inhibit the synthesis of the target protein.

In this work, we have shown the possibility of a ten-fold scaling of synthesis of AuNP-siRNA and demonstrated of their stability in different buffer solutions. The suspensions of AuNP-siRNA could be stored for a long time without losing of their colloidal stability and siRNA duplex integrity, which is the advantage of these constructions. Unfortunately, we cannot compare our results with the literature data concerning the storage problem of covalent nanoconstructions intended for the TNA delivery because such data have not been published.

## Conclusion

The synthesis of AuNP-siRNA nanoconstructions could be scaled at least to ten times without loss of physicochemical properties of the preparation. The volume increase of the reaction mixture when preparing the nanoconstructions affects the surface density of siRNA. This effect, in turn, causes changes to a similar extent in the physicochemical parameters dependent on the surface density siRNA. The noncovalent AuNP-siRNA nanoconstruction showed high colloidal stability during storage up to seven months at 4 °C. We demonstrated the formation and disintegration of the nanoparticle clusters, which were proved to be agglomerates. The noncovalent AuNP-siRNA nanoconstruction retained the surface density of siRNA and the integrity of the siRNA duplex for up to seven months of storage. The noncovalent binding of siRNA to AuNPs is a promising approach to obtain cores for multilayer nanoconstructions for the delivery of siRNA.

## Experimental

### Materials and methods

We used RNA phosphoramidite synthons, dithiothreitol (DTT), sodium citrate (Na_3_C_6_H_5_O_7_·2H_2_O, Sigma-Aldrich, USA); Cy-5.5 phosphoramidite (Primetech, Belarus); aurum hydrochloric acid (HAuCl_4_·3H_2_O, Aurat, Russia); human serum albumin (HSA, Reanal, Hungary); sodium chloride (Honeywell, Germany); formvar (SPI-Chem, USA); nutrient Dulbecco's Modified Eagle's Medium (DMEM, Life Technologies, UK). The other reactants and solvents were from Fluka (Belgium), Merck (Germany), and PanReac (Spain). Water was purified on a Simplicity 185 system (Millipore) and had a resistance of 18.2 MΩ∙cm at 25 °C. The AuNP-siRNA suspensions were prepared in vials (1.5 mL, Eppendorf safe lock tubes, #0030120086).

### Synthesis of gold nanoparticles

Gold nanoparticles were synthesized as described in a previous work [30] and a description is given in Supporting Information File 1. The resulting suspension of AuNPs (12.85 ± 0.35 nm, by TEM, and zeta potential −33.6 ± 2.0 mV) with a concentration of 3.5 × 10^−9^ M was stored at 4 °C [31].

### Synthesis of siRNA specific for GFP

The synthesis of oligoribonucleotides was carried out on an ASM-800 synthesizer (Biosset, Russia) according to the protocol of the solid-phase phosphoramidite synthesis [32]. The following oligonucleotides were synthesized: sense-GFP (5'-CAAGCUGACCCUGAAGUUCTT'), antisense-GFP (5'-GAACUUCAGGGUCAGCUUGTT), and 5’-fluorescently labeled sense-GFP-Cy5.5 (5'-Cy5.5-CAAGCUGACCCUGAAGUUCTT), which was synthesized with the use of Cy-5.5 phosphoramidite at the final stage of the synthesis. The oligonucleotides were isolated by electrophoresis in 15% polyacrylamide gel (acrylamide/*N*,*N*’-bisacrylamide (29:1) and 8 M urea) in TBE buffer (89 mM Tris, 89 mM H_3_BO_3_, and 2 mM EDTA, рН 8.3) for 1.5 h at 20 V/cm and 40 °С. The oligonucleotides were eluted from the gel with water and precipitated as the sodium salt in acetone.

The siRNA samples containing complementary oligoribonucleotides (25 µM each) and 350 mM NaCl were subjected to heating for 3 min at 95 °С, followed by cooling to 25 °С.

The samples of native siRNA (1) and fluorescently labeled siRNA (2) were synthesized.

### Noncovalent AuNP-siRNA nanoconstructions

The noncovalent AuNP-siRNA nanoconstructions were prepared in two variants.

Sample ×1: The just after synthesis AuNP suspension (135 µL) was added to native siRNA (4 µL, 25 µM). The reaction mixture was incubated for 24 h at 25 °С under stirring at 1000 rpm. Unbound siRNA was washed off with sodium citrate (1 mL, 3.88 mM;), followed by centrifugation for 30 min at 16000*g* and 25 °С. This procedure was used in all experiments for washing. The supernatant was removed and stored if necessary. The sol was diluted with sodium citrate to a volume of 5 µL.

Sample ×10: The just after synthesis AuNP suspension (1350 µL) was added to native siRNA (40 µL, 25 µM). The reaction mixture was incubated for 24 h at 25 °С under stirring at 1000 rpm. The next steps were performed as described above. The resulting sol was diluted with sodium citrate to a volume of 50 µL.

The noncovalent AuNP-siRNA nanoconstructions containing the fluorescent label were prepared similar to the native compositions using labeled siRNA (2).

Thus, we prepared the noncovalent AuNP-siRNA nanoconstructions (samples ×1 and ×10) that contained equal concentrations of the AuNP nanoparticles and siRNA molecules in different volumes of the reaction mixture.

We used equal amounts of AuNP-siRNA in all experiments and added the necessary volume of the following solutions, i.e., PBS, DMEM, 25 mM citrate buffer pH 4, 4 mM sodium citrate, 10 mM HEPES pH 7.4, 100 mM TrisHCl pH 8.3, 22 mM PB pH 4, 100 mM PB pH 7, 3% or 10% HSA in DMEM.

### Optical extinction spectra

Optical extinction spectra were recorded on a Clariostar plate fluorimeter (BMG, Labtech) in the range 400–800 nm according to the manufacturer's instructions.

### Sample storage mode

Samples ×1 were stored for eight days at 4 °C. Samples ×10 were stored for eight days, two, three, and four weeks, and seven months at 4 °C.

### Hydrodynamic characteristics of AuNP-siRNA suspensions

The hydrodynamic characteristics of the AuNP-siRNA suspensions were evaluated by the method of photon correlation spectroscopy on a Malvern Zetasizer Nano instrument (Malvern Instruments, UK). The measurements were performed at least in triplicate.

### Electrophoresis

The electrophoresis experiments in agarose or acrylamide gel were as performed as described in [16].

### Desorption of siRNA from the surface of AuNPs

The AuNP-siRNA suspension (5 µL) was incubated with 50 mM DTT (50 µL) for 30 min at 56 °С under stirring at 1000 rpm, followed by the addition of sodium citrate (1 mL). After centrifugation of the reaction mixture, the supernatant was removed, and siRNA in the supernatant was analyzed by electrophoresis in 15% native polyacrylamide gel in TBE buffer at 5 V/cm. The products on the gel were visualized by StainsAll.

### Surface density of siRNA

The surface density of siRNA in the AuNP-siRNA nanoconstructions was evaluated by the measurement of fluorescence of the labeled RNA strand on a Clariostar plate fluorimeter (BMG, Labtech) at λ_ex_ = 640 nm and λ_em_ = 700 nm [17]. The measurements were performed at least in triplicate.

### Transmission electron microscopy of AuNP-siRNA

AuNP-siRNA samples ×1 and ×10 were diluted ten times and adsorbed on formvar-coated copper grids. The excess of the liquid was removed by a pipette, and the grid was air dried. The samples were studied on a JEM 1400 TEM (Jeol, Japan) equipped with a Veleta digital camera (EM SIS, Germany) and the iTEM program, version 5.2 (EM SIS, Germany). After the addition of the HSA solution, the samples were contrasted with 2% phosphotungstic acid, pH 0.5.

The content of the AuNP-siRNA clusters in samples ×1 and ×10 was evaluated in each experimental point using the SPIP program (Image Metrology) using the digital images with a total area of 39.7 µm^2^ for each point. The number and diameter of all clusters were calculated using the particle and pore analysis module in advanced threshold regime. The clusters were distributed in diameter into several groups, i.e., <100 nm, 100–200 nm, and >100 nm.

### Statistical processing

The statistical processing of all data was performed using Student’s *t*-test with confidence probability *p* = 0.95 using Microsoft Excel.

## Supporting Information

File 1 Additional experimental data.
