# Supplementary material for: Long-term stability and scale-up of noncovalently bound gold nanoparticle-siRNA suspensions
Source: Beilstein J Nanotechnol. 2019 Dec 23;10:2568–78. doi: 10.3762/bjnano.10.248 (PMC6941443; doi:10.3762/bjnano.10.248)
Supplement: File 1 — Additional experimental data. [file Beilstein_J_Nanotechnol-10-2568-s001.pdf]

BEILSTEIN JOURNAL OF NANOTECHNOLOGY

Supporting Information

for

Long-term stability and scale-up of noncovalently bound gold nanoparticle-siRNA suspensions

Anna V. Epanchintseva, Julia E. Poletaeva, Dmitrii V. Pyshnyi, Elena I. Ryabchikova and Inna A. Pyshnaya

Beilstein J. Nanotechnol. **2019,** *10,* 2568–2578. doi:10.3762/bjnano.10.248

Additional experimental data

License and Terms: This is a supporting information file under the terms of the Creative Commons Attribution License (https://creativecommons.org/licenses/by/4.0). Please note that the reuse, redistribution and reproduction in particular requires that the authors and source are credited.
The license is subject to the *Beilstein Journal of Nanotechnology* terms and conditions: (https://www.beilstein-journals.org/bjnano)

Table S1: Values of AuNP-siRNA ζ-potential.

Sample	Peak 1, mV	Peak 1, %	Peak 2, mV	Peak 2, %	Peak 3, mV	Peak 3, %
1x	-38.9 ± 2.7	89.4 ± 5.1	-78.8 ± 10.9	10.8 ± 8.0	-96.4 ± 8.0	1.0 ± 0.5
10x	-49.4 ± 2.1	88.4 ± 6.6	-20.6 ± 8.0	8.5 ± 4.8	-90.6 ± 16.3	2.1 ± 4.4

Value of bare AuNPs ζ-potential is -33.6 ± 2.0 mV.

Table S2: Hydrodynamic parameters of AuNP-siRNA suspensions during storage at 4 °C.

Sample	Duration of storage, days	Diameter, nm[a]	PdI[a]
1x	0	33.39 ± 1.1	0.266 ± 0.025
1x	1	40.14 ± 3.17	0.322 ± 0.01
1x	8	35.35 ± 4.14	0.296 ± 0.005
10x	0	37.51 ± 1.65	0.304 ± 0.010
10x	1	36.8 ± 4.17	0.326 ± 0.006
10x	8	34.91 ± 3.31	0.3000 ± 0.005

[a] each value was calculated using at least three measurements

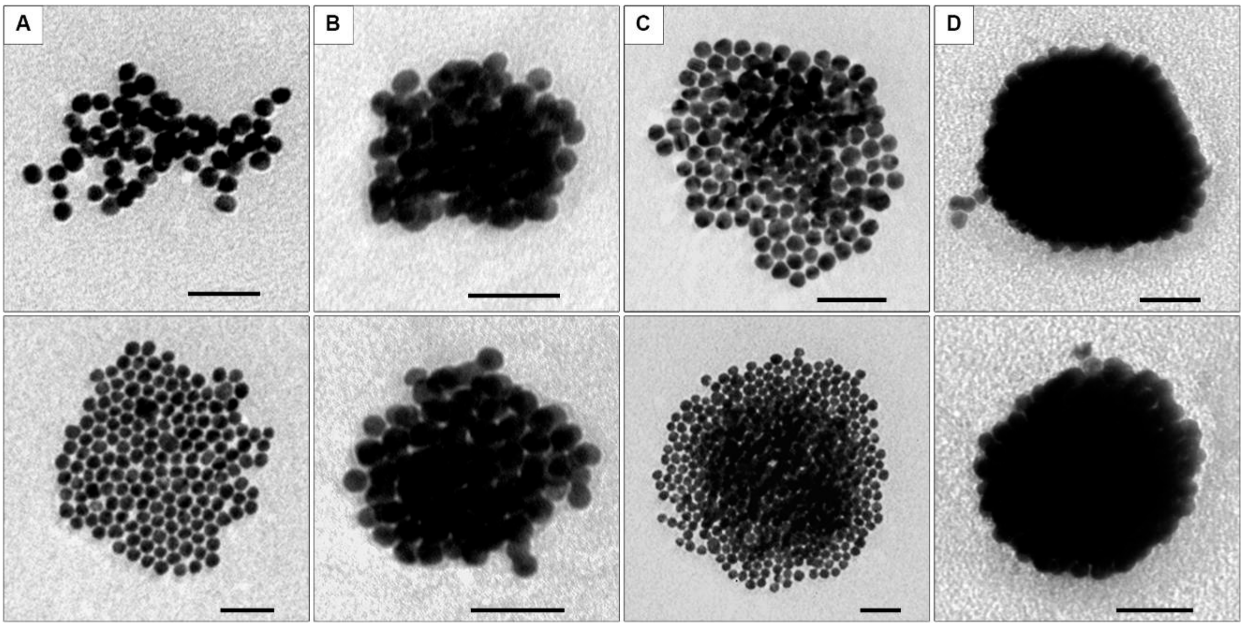

Figure S1: Different structures of AuNP-siRNA clusters observed in ×1 and ×10 AuNP-siRNA samples. All grids were prepared identically, without drying of a drop. Scale bars correspond to 50 nm.

Morphology of the clusters reflects the degree of particles clumping. Type **A -** Chains and “figures” of AuNPs-siRNA, - represent initial stages of clumping; are present in all samples. The particles are clearly separated with electron lucent space. Type **B -** Homogeneous loose rounded clusters formed by distinguishable particles, - represent more close interaction of particles. Type **C -** Rounded clusters with a multilayer core, - reflect an increase in the cluster size around the previously formed core. Type **D** - Compact rounded clusters, without peripheral single-layer zone, individual particles can be distinguished only at the periphery.

Freshly prepared and stored for one day samples x1 contained clusters of type A, and samples X10 – types A and B. Storage for eight days resulted in the formation of clusters of type C (mainly in samples x1) and type D – in samples x10.

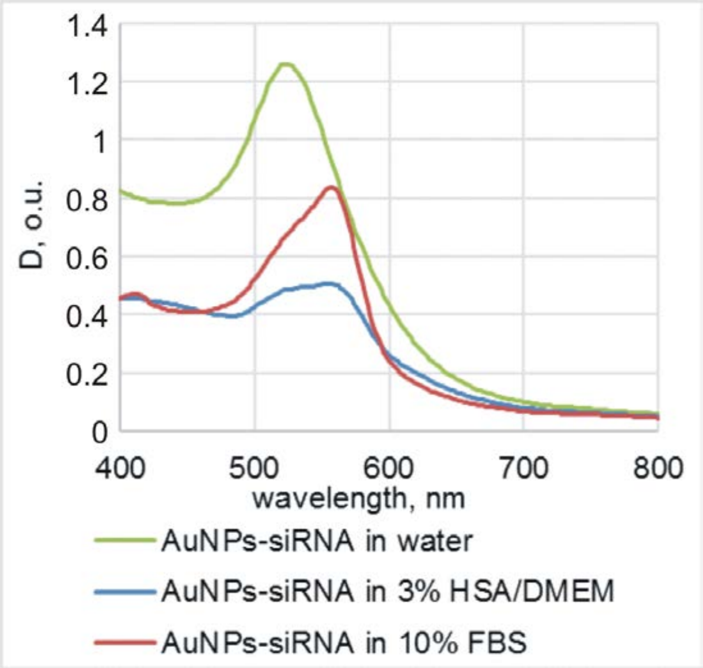

Figure S2: Optical extinction spectra of fresh AuNP-siRNA after 7 month storage after mixing with in various solutions.

Table S3: Surface density of siRNA after different storage periods of AuNPs-siRNA.

Duration of storage, days	Surface density, mol/AuNP
0	102.5 ± 3.5
1	101.3 ± 0.1
8	101.8 ± 1.1
14	101.3 ± 0.1
21	100.8 ± 0.5
28	101.8 ± 0.5

Synthesis of AuNPs was performed similarly to described in [1].

The solution of $HOC(COONa)(CH2COOONa)_2 \times 3H_2O$ (5 mL, 38.8 mM) was added under stirring to the boiled solution of $HAuCl_4 \times 3H_2O$ (45 mL, 1 mM), and the mixture was

intensively stirred for 20 min. The mixture was then cooled to room temperature, kept for 24 h, and filtered through the filter with the pore size of 45 μm.

The resulting suspension of AuNPs with a concentration of $3.5 \cdot 10^{-9}$ M was stored at 4°C, ε260 = 8.78×10^{8} M^{-1} cm–1 [2].

References

1. Frens, G. *Nature* **1973**, *24*, 20–22.
2. Liu, X.; Atwater, M.; Wang, J.; Huo, Q. *Colloids Surf. B: Biointerfaces* **2007**, *58*, 3–7.
